# Hard Tick Factors Implicated in Pathogen Transmission

**DOI:** 10.1371/journal.pntd.0002566

**Published:** 2014-01-30

**Authors:** Xiang Ye Liu, Sarah I. Bonnet

**Affiliations:** USC INRA Bartonella-tiques, UMR BIPAR ENVA-ANSES, Maisons-Alfort, France; University of Texas Medical Branch, United States of America

## Abstract

Ticks are the most common arthropod vector, after mosquitoes, and are capable of transmitting the greatest variety of pathogens. For both humans and animals, the worldwide emergence or re-emergence of tick-borne disease is becoming increasingly problematic. Despite being such an important issue, our knowledge of pathogen transmission by ticks is incomplete. Several recent studies, reviewed here, have reported that the expression of some tick factors can be modulated in response to pathogen infection, and that some of these factors can impact on the pathogenic life cycle. Delineating the specific tick factors required for tick-borne pathogen transmission should lead to new strategies in the disruption of pathogen life cycles to combat emerging tick-borne disease.

## Introduction

Ticks are the obligate blood-feeding ecto-parasites of many hosts, including mammals, birds, and reptiles, and are also vectors for several bacterial, parasitic, or viral pathogens. After mosquitoes, ticks are the second most common arthropod pathogen vector [1]. Recent intensification of human and animal movements, combined with socioeconomic and environmental changes, as well as the expanding geographical distribution of several tick species, have all contributed to the growing global threat of emerging or re-emerging tick-borne disease (TBD), along with increasing numbers of potential tick-borne pathogens (TBP) [2]. Despite an urgent requirement for in-depth information, the existing knowledge of tick pathogen transmission pathways is incomplete. *Ixodidae* possess the most complex feeding biology of all hematophagous arthropods [3], therefore the resulting difficulties in maintaining productive laboratory colonies doubtlessly explain a significant proportion of the gaps in our knowledge [4]. Moreover, because of the disadvantages of current TBD control methods (resistance, environmental hazard, increased cost), new approaches are urgently needed. Among these, vaccine strategies targeting those molecules that play key roles in vector competence are particularly promising [5], [6]. Consequently, research on molecular interactions between ticks and pathogens as well as the identification of suitable antigenic targets is a major challenge for the implementation of new TBD control strategies.

**Figure 1:**
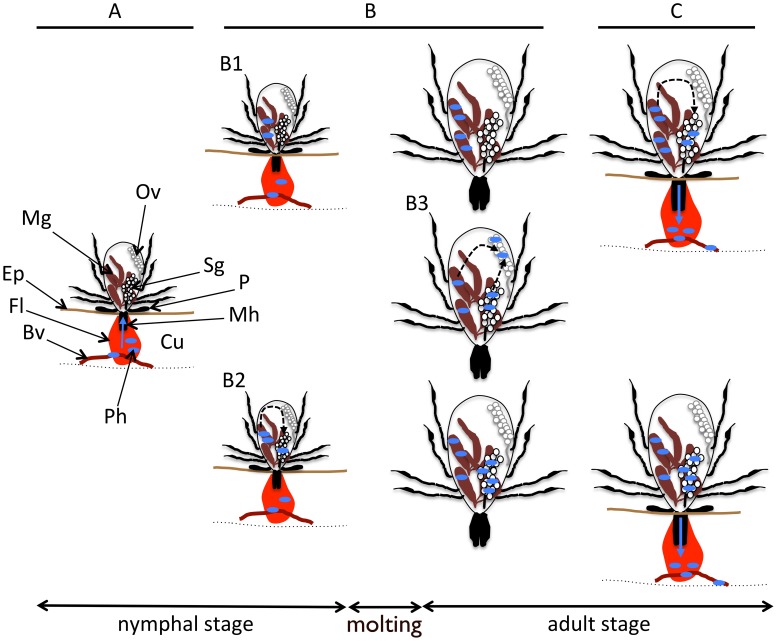
Possible TBP transmission route from an infected host to a new host, via hard ticks. Note that pathogen multiplication can occur in both the tick midgut or salivary glands, depending on the pathogen. Arrows indicate migrating pathogen pathways. A: Acquisition of TBP by a nymphal stage tick during blood feeding. B: TBP development within the tick; preservation in the tick gut (B1); dissemination into the hemolymph and migration to the salivary glands, which can occur either immediately after acquisition (B2) or after the stimulus of a new blood meal (C); dissemination into the hemolymph and migration to the ovaries (B3), which may or may not occur, and which can lead to transovarial transmission and infection of the succeeding generation. C: TBP transmission from the subsequent adult tick stage to a new vertebrate host during blood feeding; BV: blood vessel; CU: cutis; EP: epidermis; FL: feeding lesion; MG: midgut; MH: mouthparts (chelicera and hypostome); OV: ovaries; P: palp; TBP: tick-borne pathogens; SG: salivary glands. Small blue ovals represent TBP.

During the blood feeding process, ticks confront diverse host immune responses and have evolved a complex and sophisticated pharmacological armament in order to successfully feed. This includes anti-clotting, anti-platelet aggregation, vasodilator, anti-inflammatory, and immunomodulatory systems [7]. For most TBP, transmission via the saliva occurs during blood feeding (Figure 1) and such tick adaptations may promote TBP transmission, notably by interfering with the host immune response 8–10. Moreover, during their development within the tick and their subsequent transmission to the vertebrate host, pathogens undergo several developmental transitions and suffer population losses, to which tick factors presumably contribute. Several studies have clearly reported that pathogens can influence tick gene expression, demonstrating molecular interaction between the vector and pathogen 11–24. Our review briefly outlines TBP transmission, highlights evidence of molecular interactions between hard ticks and TBP, and describes several tick molecules implicated in pathogen transmission.

## Tick-Borne Pathogen Transmission

Hard ticks progress through larval, nymphal, and adult stages, all of which require a blood meal. For the majority of hard ticks of medical and veterinary relevance (including *Ixodes* spp., *Dermacentor* spp., and *Amblyomma* spp.), a three-stage life cycle including host seeking, feeding, and off-host molting (or egg laying), is the most common developmental pattern, whereas some ticks, such as *Rhipicephalus microplus* (formerly *Boophilus microplus*) undergo a single-host cycle. Ticks feeding on a pathogen-infected vertebrate host also imbibe these pathogenic microorganisms, and, once ingested, the pathogen's life cycle differs depending on the pathogen (Figure 1). In the midgut, pathogens such as *Anaplasma marginale* can undergo initial multiplication within membrane-bound vacuoles [25], [26]. *Borrelia* spp. or *Bartonella* spp. remain in the midgut during tick molting and only invade the salivary glands after a new blood meal stimulus [27], [28], whereas *Babesia* spp. and *Rickettsia* spp. immediately invade both the tick ovaries and salivary glands via the hemolymph [29], [30]. *Theileria* spp. parasites exhibit a similar cycle in the vector but without ovarian invasion [31]. *Anaplasma* spp. and some arboviruses also migrate from the gut to salivary glands where they remain during molting, up until the next tick life stage and blood feeding episode [32], [33]. Once inside the tick, intestinal, salivary, or ovarian barriers must be crossed, and multiple distinct cell types must be invaded for pathogenic multiplication to occur. During tick infection and transmission, TBP must also adapt to tick-specific physiological and behavioral characteristics, particularly with regard to blood feeding, blood meal digestion, molting, and immune responses [34]. Finally, pathogens are re-transmitted to new vertebrate hosts during tick blood feeding via the saliva and, and for certain pathogens, they can be transferred to the next tick generation via transovarial transmission (Figure 1). This vertical transmission is an absolute necessity for those TBP infecting single-host tick species such as the *R. microplus*-transmitted *Babesia bovis*.

## Functional Transcriptomic/Proteomic Studies of Tick and Tick-Borne Pathogen Interactions

Several investigations performed in different models with varying approaches are summarized in Table 1. In general, they report that tick gene or protein expression can be regulated in response to pathogen infection. Most of the modulated transcripts or proteins were not associated with a known protein or an assigned function; however, some were able to be annotated as putative proteins.

**Table 1:** Functional transcriptomic/proteomic tick and TBP interaction studies.

Tick species	Tick organs	Tick-borne pathogens	Technique used	Number of differentially expressed transcripts/proteins	Refs
**Transcriptomic studies**					
*D. variabilis* female	SG, MG, OV	*R. montanensis*	DD-PCR	54	[11]
*I. scapularis* nymph	SG	*B. burgdorferi*	LCS	10	[14]
*I. scapularis* nymph	WT	*Langat virus*	MH	48	[17]
*I. scapularis* embryos	IDE8 tick cells	*A. marginale*	SSH	35	[15]
*I. ricinus* female	WT	*B. burgdorferi*	SH	11	[13]
*R. appendiculatus* female	SG	*T. parva*	LCS	3	[12]
*R. microplus* male	SG	*A. marginale*	SSH	99	[16]
**Proteomic studies**					
*I. scapularis* embryos	IDE8 tick cells	*A. marginale*	2D-DIGE, MALDI-TOF MS	3	[15]
*I. scapularis* embryos	ISE6 tick cells	*A. phagocytophilum*	IEF, 2D-DIGE, MALDI-TOF MS, RP-LC MS/MS	5	[20]
*R. bursa* female	WIO	*T. annulata*	2D-DIGE, RP-LC MS/MS, MALDI-TOF MS	16	[21]
*R. microplus* female	OV	*B. bovis*	IEF, 1/2DGE, HPLC-ESI-MS/MS	19	[18]
*R. microplus* female	MG	*B. bovis*	IEF, 1/2DGE, HPLC-ESI-MS/MS	20	[19]
*R. sanguineus* female	WIO	*Ric. conorii*	2D-DIGE, RP-LC MS/MS, MALDI-TOF MS	10	[21]
*R. sanguineus* female	WIO	*E. canis*	2D-DIGE, RP-LC MS/MS, MALDI-TOF MS	6	[21]
*R. turanicus* female	WT	*A. ovis*	IEF, 2D-DIGE, MALDI-TOF MS, RP-LC MS/MS	50	[20]
*R. turanicus* female	WIO	*A. ovis*	2D-DIGE, RP-LC MS/MS, MALDI-TOF MS	9	[21]

SG: salivary glands, MG: midgut, OV: ovaries, WT: whole ticks, WIO: whole internal organs; DD-PCR: differential-display polymerase chain reaction, LCS: cDNA library clones sequencing, MH: microarray hybridization, SH: subtractive hybridization, SSH: suppression-subtractive hybridization; D: dimensional, DIGE: differential in-gel electrophoresis, DGE: dimensional gel electrophoresis, ESI: tandem electrospray, HPLC: high-performance liquid chromatography, IEF: isoelectric focusing, MALDI-TOF: matrix-assisted laser desorption/ionization time-of-flight, MS: mass spectrometry, RPLC: reversed phase liquid chromatography.

### Transcriptomic studies

Macaluso et al. used differential-display polymerase chain reaction (DD-PCR) to identify *Dermacentor variabilis* tick transcripts, which were variably expressed in response to *Rickettsia montanensis* infection [11]. Among identified transcripts, nine were down-regulated in the infected tick midgut; five transcripts (clathrin-coated vesicle ATPase, peroxisomal farnesylated protein, α-catenin, salivary gland protein SGS-3 precursor, and glycine-rich protein) were also down-regulated in the tick salivary glands; whereas six (clathrin-coated vesicle ATPase, peroxisomal farnesylated protein, Ena/vasodilator-stimulated phosphoprotein-like protein, α-catenin, tubulin α-chain, and copper-transporting ATPase) were up-regulated in infected tick ovaries. However, it was clearly demonstrated that the DD-PCR technique poses serious problems in the re-amplification of selected transcripts and generates many false positives [35]; consequently, this method is rarely used today.

EST (Expressed Sequence Tag) sequences derived from cDNA libraries have also been used to analyze and compare gene expression in *Rhipicephalus appendiculatus* ticks infected with *Theileria parva*. Results suggested an up-regulation in the expression of some glycine-rich proteins named TC1268, TC1278, and TC1272, in infected salivary glands [12].

Subtractive hybridization libraries have also been used in order to investigate the response of *Ixodes ricinus* whole ticks to blood feeding and to infection with *Borrelia burgdorferi*, the agent for Lyme disease [13]. This study showed that 11 genes were specifically induced after a blood meal on *B. burgdorferi*-infected guinea pigs, which included several thioredoxin peroxidases, glutathione S-transferase, and defensins.

The response to *A. marginale* infection was also analyzed in male *R. microplus* salivary glands by subtractive hybridization libraries [16]. Based on EST sequences, 43 unique transcripts (such as proline- or glycine-rich proteins) were up-regulated, whereas 56 were down-regulated (including histamine binding protein, immunoglobulin G binding protein, or the Kunitz-like protease inhibitor).

When analyzing the response of *Ixodes scapularis* nymphal ticks to *B. burgdorferi* infection via the sequencing of cDNA library clones, Ribeiro et al. showed that ten salivary gland genes were significantly differentially expressed during bacterial infection [14]. Among these ten genes, seven were overrepresented in the *B. burgdorferi* infected nymphs, including those coding for the 5.3-kDa peptide family, basic tail family, and histamine-binding protein (HBP) family; however, three genes coding for HBP family proteins were overexpressed in the non-infected nymphs.

To investigate the effect of feeding and flavivirus infection on the salivary gland transcript expression profile in *I. scapularis* ticks, a first-generation microarray was developed using ESTs from a salivary gland-derived cDNA library [17]. Among the 48 salivary gland transcripts presenting differential expression after virus infection, three were statistically differentially regulated during the three analyzed post-feeding periods, two were up-regulated, and one was down-regulated. One of the up-regulated genes belonged to the 25-kDa salivary gland protein family presenting homology to lipocalins, whose function is the transportation of small molecules.

Finally, several differentially regulated genes were identified by using suppression-subtractive hybridization analyses of cultured IDE8 *I. scapularis* tick cells in response to *A. marginale* infection [15]. Twenty-three genes were up-regulated, including glutathione S-transferase, vATPase, or selenoprotein W2a; whereas six were down-regulated (including β-tubulin, ferritin, or R2 retrotransposon reverse transcriptase-like protein).

All approaches used in the above-mentioned studies led to the identification of differentially expressed tick transcripts in response to TBP infection. Some of the observed discrepancies between models may be due both to the models themselves and to the differing sensitivity of specific techniques. In future, transcriptomic analysis may be performed by using new powerful NGS techniques that harbor high sensitivity. Moreover, using the same technique to analyze transcripts in *A. marginale*-infected IDE8 tick cells [15], [16] and *A. marginale*-infected *R. microplus* demonstrated that more differentially regulated transcripts were identified in vivo (Table 1), suggesting that in vitro models should be used with caution. In any case, the lack of genomic information for almost all tick species leads to difficulties in data analysis. The analysis of mRNA expression levels is undoubtedly an effective method to identify tick gene expression during TBP infection, but the level of mRNA and the concentration of corresponding proteins only have a correlative, rather than a causative, association. Therefore, the quantities of translated proteins in ticks in response to TBP infection should also be assessed.

### Proteomic studies

Proteomic profiling of *B. bovis*-infected *R. microplus* ticks demonstrated that ten proteins were differentially up-regulated in ovaries, including endoplasmic reticulum protein, glutamine synthetase, and a family of Kunitz-type serine protease inhibitors, and nine proteins were down-regulated, including tick lysozyme and a hemoglobin subunit [18]. In the midgut, 15 proteins were up-regulated, including gamma-glutamytransferase1 and a putative ATP synthase-like protein; five proteins were down-regulated, including heat shock cognate 70 protein, putative heat shock-related protein, and signal sequence receptor beta [19].

The proteomic profile of *I. scapularis* embryonic tick cells was investigated in response to *Anaplasma* spp. infection [15], [20]. Results showed that the translation elongation factor 1γ was up-regulated, whereas GST (glutathione-S-transferase) and a putative high-mobility group-like protein were under-expressed in *A. marginale*-infected IDE8 tick cells [15]. HSP70 (heat shock protein 70) was over-expressed, but other putative HSPs were under-expressed in *Anaplasma phagocytophilum* infected ISE6 tick cells [20].

Differentially expressed proteins were also identified in *Rhipicephalus* spp. ticks infected with *Anaplasma ovis*, *Theileria annulata*, *Rickettsia conorii*, or *Erhlichia canis* by comparing them with non-infected ticks [20], [21]. Results showed that the protein expression profile (among which actin, enolase, or guanine nucleotide-binding protein were identified) varied according to the analyzed models. Fifty-nine proteins have been identified as differentially expressed in *A. ovis*-infected *Rhipicephalus turanicus* ticks, 16 in *T. annulata*-infected *Rhipicephalus bursa*, ten in *Ric. conorii*-infected *Rhipicephalus sanguineus*, and six in *E. canis*-infected *R. sanguineus*.

Thus, relatively few studies have focused on the proteome, reflecting the relative difficulty of studying the subject compared to research on transcripts. However, analyzing protein expression allows one to take into account any translational modifications that may occur.

## Tick Factors Implicated in Tick-Borne Pathogen Transmission

As reported above, the expression of some tick factors can be modulated by TBP infection during stages of acquisition, multiplication/migration in the vector, and/or transmission to hosts. These factors correspond to two types of molecules: those facilitating pathogen development, and those which limit it, i.e., the molecules from the tick's own immune system. However, based on the aforementioned studies, it is difficult to confirm whether the identified molecules are specific to the studied microorganisms. Therefore, functional studies are required to validate their implication in pathogen development. Antibodies can be used for this purpose, but the most widely used method currently is RNA interference (RNAi), a gene-silencing technique suited to tick analysis when other methods of genetic manipulation are rare [36]. Tick factors that have been identified as implicated in TBP life cycles are summarized in Table 2 and described below.

**Table 2:** Hard tick factors, which contribute to/inhibit TBP acquisition, multiplication and migration, and transmission.

Tick species	Tick factors	Genbank accession number	Tick-borne pathogens	Expression level in pathogen infected ticks	Pathogen life cycle modified	Refs
*D. variabilis*	GST	DQ224235	*A. marginale*	Up-regulation	Acquisition, multiplication	[15], [51]
	Subolesin	AY652657	*A. marginale*	Up-regulation	Acquisition, transmission	[46], [47], [51]
	varisin	AY181027	*A. marginale*	Down-regulation (MD), Up-regulation (SG)	Acquisition, multiplication	[44]
	vATPase	ES429091	*A. marginale*	Up-regulation	Acquisition	[15], [51]
	SelM	ES429105	*A. marginale*	Up-regulation	Multiplication	[15], [51]
*I. scapularis*	P11	DQ066011	*A. phagocytophilum*	Up-regulation	Acquisition, migration	[57]
	Salp15	AF209914	*B. burgdorferi*	Up-regulation	Transmission	[22], [58]
	Salp16	AF061845	*A. phagocytophilum*	Up-regulation	Acquisition	[38]
	Salp25D	AF209911	*B. burgdorferi*	No change	Acquisition	[22], [42]
	Subolesin	AY652654	*A. phagocytophilum*	No change	Acquisition	[47], [49]
	Subolesin	AY652654	*B. burgdorferi*	Unknown	Acquisition, transmission	[50]
	tHRF	DQ066335	*B. burgdorferi*	Up-regulation	Transmission	[62]
	TROSPA	AY189148	*B. burgdorferi*	Up-regulation	Multiplication	[23]
	TRE31	HQ998856	*B. burgdorferi*	Up-regulation	Migration	[24]
	TSLPI	AEE89466	*B. burgdorferi*	Up-regulation, then down-regulation	Acquisition, transmission, multiplication	[53]
	α1, 3-fucosyltransferases	_XM_002401196	*A. phagocytophilum*	Up-regulation	Acquisition	[52]
		_XM_002404622				
		_XM_002406085				
		_XM_002415522				
*R. microplus*	Subolesin	DQ159966	*A. marginale, B. bigemina*	Up-regulation	Acquisition	[46], [48]
*D. variabilis*	DvKPI	EU265775	*R. montanensis*	Up-regulation	Acquisition	[68], [69]
*I. scapularis*	5.3-kD protein	EEC00268	*A. phagocytophilum*	Up-regulation	Acquisition and transmission	[67]

SG: salivary glands, MG: midgut.

## Tick Factors Contributing to Tick-Borne Pathogen Acquisition

The host skin site, to which the tick attaches during feeding, is a critical interface between ticks, hosts, and the TBP [37]. For ticks, it is the location of their indispensable blood meal; for hosts, it acts as the barrier preventing blood loss and pathogen invasion; however, for pathogens, it is an ecologically privileged niche that should be exploited.

Salp16, an *I. scapularis* salivary protein, facilitates *A. phagocytophilum* acquisition [38]. In Salp16-deficient ticks, infection of tick salivary glands by *A. phagocytophilum* is strongly decreased. Interestingly, silencing Salp16 does not affect *B. burgdorferi* acquisition, indicating pathogen specificity [38]. Salp16 is implicated in vertebrate host blood-cell membrane digestion, facilitating the escape of *A. phagocytophilum* from host-cell vacuoles and then its subsequent dissemination throughout the tick's body, including salivary glands [39], [40].

Salp25D, an antioxidant protein identified in both the midgut and salivary glands of *I. scapularis*, is up-regulated following blood meals [41], [42]. Injecting Salp25D-specific dsRNA into the tick body silences Salp25D salivary gland expression and impairs *B. burgdorferi* acquisition. However, silencing midgut Salp25D expression by injecting dsRNA into the tick anal pore does not impact on *B. burgdorferi* acquisition, suggesting that the same protein may play different roles according to the organ concerned [42].

Defensins are components of the tick's innate immune system, protecting ticks from both gram-negative and gram-positive bacteria [43]. Accordingly, defensins are up-regulated in *R. montanensis*-infected *D. variabilis* [43]. Interestingly, varisin, a specific *D. variabilis* defensin, is also over-expressed in *A. marginale*-infected tick salivary glands, but is under-expressed in the midgut after feeding on pathogen-infected sheep, suggesting that *A. marginale* might down-regulate varisin expression to establish gut infection [44]. Silencing varisin expression via RNAi was predicted to increase tick bacterial infection levels. However, silencing produced the opposite result, as levels of *A. marginale* were significantly reduced in tick midgut after feeding on an infected calf [44].

Subolesin, another tick protective molecule discovered in *I. scapularis* [45], was proven to be up-regulated in *A. marginale*-infected ticks [46]. Either gene silencing or immunization with a subolesin recombinant protein results in lower *A. marginale*, *A. phagocytophilum*, and *Babesia bigemina* infection levels in hard ticks, demonstrating no TBP species specificity [47]–[49]. In addition, oral vaccination of mice with vv-sub (vaccinia virus-expressed subolesin) reduces *B. burgdorferi* acquisition by *I. scapularis* larval ticks from infected mice and *B. burgdorferi* transmission to uninfected mice, as well as numbers of ticks that have fully engorged [50]. Consequently, subolesin not only plays an important role in the acquisition and transmission of several pathogens, but also contributes to effective tick blood feeding. The correlation between tick subolesin expression and pathogen infection highlights subolesin's role in innate tick immune responses [51]. Alternatively, subolesin could up-regulate factors facilitating tick pathogen acquisition. Indeed, inhibiting subolesin expression results in lower pathogen infection levels, which could perhaps be influenced by other molecular pathways such as those required for gut and salivary gland function and development, resulting in the ingestion of less infected blood [48]. On the other hand, such inhibition may suppress the expression of other subolesin-regulated genes required for pathogen infection and multiplication [46].

During *A. phagocytophilum* acquisition by *I. scapularis*, α1,3-fucosyltransferases expression is up-regulated in ticks [52]. Silencing three α1,3-fucosyltransferases in *I. scapularis* nymphs significantly decreases *A. phagocytophilum* acquisition from infected mice, but not tick engorgement and bacteria transmission from infected ticks to mice [52]. This strongly suggests that *A. phagocytophilum* modulates α1,3-fucosyltransferase expression and utilizes α1,3-fucose to colonize ticks during acquisition.

At the tick bite site, a strong innate immune response is initiated by the host's complement cascade [8]. Schuijt et al. discovered that TSLPI (tick salivary lectin pathway inhibitor) interferes with the human lectin complement cascade, leading to decreased *Borrelia* lysis [53]. They suggest that TSPLI could play a crucial role in successful acquisition of *Borrelia* by *I. scapularis* from *Borrelia*-infected hosts. When pathogen-free *I. scapularis* larvae were engorged on *B. burgdorferi*-infected mice, which had been immunized with recombinant TSLPI protein, *Borrelia* acquisition by the larval ticks was effectively impaired, strengthening TSLPI's predicted role [53].

Silencing putative GST (glutathione S-transferase) and vATPase (H^+^ transporting lysosomal vacuolar proton pump) genes in *D. variabilis* ticks inhibits *A. marginale* infection after tick feeding on infected calves [51]. It was hypothesized that GST may protect tick gut cells from oxidative stress caused by *A. marginale* infection, and vATPase might facilitate *A. marginale* infection in tick gut and salivary glands by receptor-mediated endocytosis.

## Tick Factors Contributing to Tick-Borne Pathogen Multiplication or Migration within Ticks

The tick midgut is the first major defensive barrier against pathogen infection [54], [55]. In order to first establish an infection and then promote transmission, pathogens need to be able to successfully overcome this barrier (by colonizing cells or by passing through or between cells) [56]. Pathogens imbibed during the blood meal must contend with heterophagic blood meal digestion, escape the midgut, and then migrate via the hemolymph to the salivary glands, where a second round of multiplication often occurs, culminating during transmission feeding and often dependent upon resumption of tick feeding. Following multiplication, TBP are transmitted via the saliva to the new host; the efficiency of this process can be influenced by the replication level [56]. These complex migration/multiplication processes are sure to require diverse molecular interactions between the TBP and the vector.

To date, only the tick protein TROSPA (tick receptor outer surface protein A), identified in *I. scapularis* ticks infected with *B. burgdorferi*, is thought to influence the TBP life cycle in the midgut [23]. TROSPA is a specific ligand for *B. burgdorferi* OspA and is required for successful spirochetes colonization of the tick midgut [23]. Blocking TROSPA with antisera, or silencing TROSPA expression via RNAi, reduced the ability of *B. burgdorferi* to adhere to the tick gut in vivo, thereby preventing efficient colonization of the vector and reducing pathogen transmission to the mammalian host [23].

The TRE31 *I. scapularis* tick gut protein is involved in *B. burgdorferi* migration from tick midgut to salivary glands [24]. Knocking down TRE31 expression by directly injecting TRE31-dsRNA into the gut of *B. burgdorferi*-infected *I. scapularis* nymphs results in unchanged numbers of gut *B. burgdorferi*, but significantly fewer spirochetes in tick hemolymph and salivary glands [24], suggesting that TRE31 likely enables spirochetes migration from tick midgut to salivary glands. Interestingly, it was demonstrated that *B. burgdorferi* outer-surface lipoprotein BBE31 can interact with TRE31, and that anti-BBE31 antibodies also decrease numbers of *Borrelia* entering the hemolymph [24].

P11, an *I. scapularis* salivary gland secreted protein, is up-regulated in response to *A. phagocytophilum* infection and facilitates migration of *A. phagocytophilum* from tick midgut to salivary glands [57]. Silencing P11 effectively impairs *A. phagocytophilum* infection of tick haemocytes in vivo and, consequently, decreases pathogen infection levels both in haemolymph and in salivary glands [57]. P11 is thought to enable haemocyte infection by *A. phagocytophilum*, permitting pathogen dissemination into the tick body [57].

Silencing *D. variabilis* tick GST and SelM (salivary selenoprotein M) genes showed that *A. marginale* multiplication was inhibited in salivary glands after tick TBP acquisition from infected calves [51]. *A. marginale* may increase GST and SelM expression to reduce oxidative stress caused by pathogen infection that may help pathogen multiplication in tick cells.

Finally, the *I. scapularis* protein TSLPI previously mentioned is also thought to be implicated in spirochetal multiplication within ticks [53]. Indeed, when some larvae were fed on *Borrelia*-infected mice passively immunized with rTSPLI antiserum, the succeeding nymphal stage had lower spirochetal loads than the control group [53].

## Tick Factors Contributing to Tick-Borne Pathogen Transmission to Vertebrate Hosts

In most transmission cases, pathogens present in tick salivary gland cells invade vertebrate hosts at the skin site where ticks have salivated during blood feeding [8]. Some factors present in the saliva are then used by microorganisms to increase their pathogenicity and evade host immune responses [8]–[10]. A few of these factors have been identified and are listed below.

Salp15 is a salivary gland protein expressed by both *I. scapularis* and *I. ricinus* ticks during engorgement [41], [58]. During blood feeding, *B. burgdorferi* induces and usurps Salp15 to facilitate murine infection [22]. Silencing Salp15 in *I. scapularis* drastically reduces the capacity of *B. burgdorferi* to infect mice [22]. Salp15 affects T-cell proliferation by binding to the CD4 (+) co-receptor [59] and inhibits dendritic cell activation by binding to the C-type lectin DC-SIGN [60]. When binding to *B. burgdorferi* outer surface protein C (OspC) [22], Salp15 protects the bacteria from antibody-mediated killing and inhibits keratinocyte inflammation [61].

*I. scapularis* tick histamine release factor (tHRF) also contributes to tick engorgement and host-transmission of *B. burgdorferi* [62]. Silencing tHRF by RNAi significantly decreases *B. burgdorferi* burden in mice heart and joints and markedly impairs tick feeding. Moreover, the *B. burgdorferi* tick burden is substantially lower in *I. scapularis* fed on tHRF antiserum-immunized mice, and the spirochete burden is markedly reduced in these mice [62].

During the rapid tick-feeding phase, tick sensitivity to histamine declines [63], [64], and expression of HBPs (histamine binding proteins) decreases from 48 to 72 hours post-tick attachment, whereas tHRF increases from 0 to 48 hours post-tick attachment [62]. It has been speculated that the reciprocal expression of HBPs and tHRF may augment local histamine concentration at the tick-feeding site during the rapid feeding phase, thereby modulating vascular permeability and enhancing blood flow, which in turn facilitates tick engorgement [62]. Moreover, the vasodilatory effect of histamine might contribute to the efficient dissemination of *Borrelia* from the original tick-feeding site to distal sites [62].

To determine TSPLI's role in *B. burgdorferi* transmission from tick to host, TSLPI-dsRNA was injected into *B. bugdorferi*-infected *I. scapularis* nymphs, or rTSLPI rabbit antiserum was used to immunize mice [53]. *Borrelia* transmission to mice was impaired via TSLPI-silenced nymphs, as well as from nymphs to rTSLPI antiserum-immunized mice, demonstrating that TSLPI plays a significant role in the transmission of *Borrelia* from arthropod vectors to vertebrate hosts [53]. Indeed, in each case, the spirochete burden was significantly lower after seven days in mice skin and heart, and after 21 days in mice joints. It is known that both classical and alternative complement pathways are involved in complement-dependent killing of *Borrelia* [65]. Schuijt et al. demonstrated that TSLPI inhibits direct killing of *B. burgdorferi* by the complement system and inhibits phagocytosis of *B. burgdorferi* by human neutrophils, as well as *Borrelia*-induced complement-mediated chemotaxis, by directly inhibiting the activation of the MBL (mannose-binding lectin) complement pathway [53].

## Tick Factors Inhibiting Tick-Borne Pathogen Acquisition and Transmission

An *I. scapularis* salivary gland gene family encoding 5.3-kD proteins, which are up-regulated by the tick signaling transducer activator of transcription (STAT) pathway and by *A. phagocytophilum* infection, might belong to a novel antimicrobial peptide (AMP) gene family [66], [67]. When silencing a member of 5.3-kD protein gene family (gene-15), the *A. phagocytephilum* infection of tick salivary glands and transmission to mammalian host were significantly increased [67]. Therefore, the salivary gland gene family encoding 5.3-kD proteins is involved in anti-*A. phagocytophilum* defense. It is the only reported tick factor which can inhibit both tick-borne pathogen acquisition and transmission. This function probably contributes to its regulation by the tick's STAT pathway, which also plays a role in controlling *A. phagocytophilum* infection in ticks and transmission to the host [67].

Finally, one *D. variabilis* kunitz protease inhibitor (DvKPI) was found to be up-regulated both by blood feeding and *Rickettsia montanensis* infection [68]. When silencing DvKPI, the bacterial colonization of tick midgut was increased to 90% [69], suggesting that this molecule can limit *R. montanensis* acquisition by ticks, possibly by limiting bacterial host cell invasion.

## Conclusion

The interactions existing between ticks and tick-borne pathogens are complex. Interacting tick factors function in a finely tuned equilibrium to influence pathogen transmission. Several tick immune factors impede pathogen expansion, whereas some factors promote pathogen infection during their transmission from one infected host to another. It is now firmly established that tick-borne pathogen infection induces differential expression of tick genes. However, a global analysis both at the transcriptional or protein levels, similar to those presented in this review, does not enable us to differentiate whether tick responses are due to a specific pathogen that has co-evolved with the tick, or whether such tick responses may belong to an innate immune response to any invading organism. Moreover, genes that are thought to be regulated during pathogen development need to be confirmed with functional studies. Therefore, with the development of newer and more efficient biological techniques, such as RNAi, we expect rapid progress in the elucidation of the molecular mechanisms governing pathogen transmission by ticks.

Delineating the specific pathogen and tick ligands required for TBP acquisition, development, and transmission should lead to the development of new TBP-targeting strategies. Such factors could become candidates for anti-tick and anti-TBP vaccines, providing novel approaches to preventing tick-borne diseases. Indeed, in light of our limited understanding of immunity to TBP, TBP strain diversity, and more generally the transmission of multiple TBP by the same tick species, vaccine strategies that target conserved tick components playing key roles in vector infestation and vector capacity have become particularly attractive [5]. Anti-tick vaccines based on recombinant antigens are environmentally safe, are less likely to cause selection for resistant strains compared to acaricides, and can incorporate multiple antigens to target a broad range of tick species and their associated TBP [6]. Anti-tick vaccines could potentially indirectly reduce TBD transmission by reducing the tick burden, or directly, through interference with tick components that enhance TBP transmission. For vaccines acting indirectly, reduction in tick burden is unlikely to be achieved unless the targeted tick species feeds principally on the host species for which the vaccine is intended. While this holds true for *R. microplus* and cattle [70], it does not for several species of ticks responsible for important TBD, such as *Ixodes* spp., for which a direct effect on transmission must be sought.

**Key Learning Points**

- The route of tick-borne pathogens from an infected vertebrate host to a new host via hard ticks is composed of three major steps: (1) acquisition of the pathogen by ticks, (2) pathogen expansion and movement within ticks, and (3) pathogen transmission from an infected tick to a vertebrate host.
- The expression of some tick factors can be modulated in response to pathogen infection, and these factors can impact on the pathogenic life cycle.
- Tick factors contributing to tick-borne pathogen transmission are potential vaccine candidates for controlling tick-borne disease.

**Five Key Papers in the Field**

1. McNally KL, Mitzel DN, Anderson JM, Ribeiro JM, Valenzuela JG, et al. (2012) Differential salivary gland transcript expression profile in *Ixodes scapularis* nymphs upon feeding or flavivirus infection. Ticks Tick Borne Dis 3: 18–26.
2. Rachinsky A, Guerrero FD, Scoles GA (2007) Differential protein expression in ovaries of uninfected and *Babesia*-infected southern cattle ticks, *Rhipicephalus (Boophilus) microplus*. Insect Biochem Mol Biol 37: 1291–1308.
3. Ramamoorthi N, Narasimhan S, Pal U, Bao F, Yang XF, et al. (2005) The Lyme disease agent exploits a tick protein to infect the mammalian host. Nature 436: 573–577.
4. Pal U, Li X, Wang T, Montgomery RR, Ramamoorthi N, et al. (2004) TROSPA, an *Ixodes scapularis* receptor for Borrelia burgdorferi. Cell 119: 457–468.
5. Dai J, Narasimhan S, Zhang L, Liu L, Wang P, et al. (2010) Tick histamine release factor is critical for *Ixodes scapularis* engorgement and transmission of the lyme disease agent. PLOS Pathog 6: e1001205. doi:10.1371/journal.ppat.1001205
